# Ordinal Regression Research Based on Dual Loss Function—An Example on Lumbar Vertebra Classification in CT Images

**DOI:** 10.3390/diagnostics15232949

**Published:** 2025-11-21

**Authors:** Chia-Pei Tang, Hong-Yi Chang, Yu-Ming Hsu, Tu-Liang Lin

**Affiliations:** 1 Division of Gastroenterology, Department of Internal Medicine, Dalin Tzu Chi Hospital, Buddhist Tzu Chi Medical Foundation, Chiayi 622401, Taiwan; 2 School of Medicine, Tzu Chi University, Hualien City 970374, Taiwan; 3 Department of Management Information System, National Chiayi University, Chiayi City 60054, Taiwan

**Keywords:** ordinal regression, MobileNet-v3-Large, deep learning, lumbar spine classification, sarcopenia

## Abstract

**Background/Objectives**: Some classification problems involve ordered categories (e.g., low–medium–high), which are better modeled as ordinal regression. This study aimed to propose and evaluate a dual loss framework—Ordinal Residual Dual Loss—for lumbar vertebra classification on CT images to assist L3 identification and sarcopenia detection. **Methods**: In this retrospective study, lumbar spine CT images were used to train a deep learning model based on a MobileNet-v3-Large network. The proposed framework combines standard cross-entropy loss for classification with an Ordinal Residual Loss defined on the difference between output probabilities and target ordinal probabilities. **Results**: Experimental results show that the Ordinal Residual Dual Loss approach outperforms using cross-entropy alone and also surpasses methods from previous studies in lumbar vertebra classification performance. **Conclusions**: Leveraging a dual loss design that incorporates ordinal information improves vertebral level classification on CT images and has potential to support more accurate automated L3 localization and sarcopenia assessment in clinical practice.

## 1. Introduction

In image classification tasks, many real-world prediction problems involve ordinal relationships between categories. For example, in age estimation from facial images, the ages may be ordered from 1 to 99 years; or in medical diagnosis, the severity of a disease can be categorized as mild, moderate, or severe. These are problems of ordinal regression. Although common classification loss functions, such as cross-entropy loss, can be used, they do not take into account the ordinal relationships between classes. For instance, the loss incurred by predicting a mild condition for a patient with a severe condition should be greater than the loss for predicting a moderate condition, since the difference between mild and severe is greater than that between moderate and severe. We hypothesize that in ordinal classification, pairing cross-entropy with an ordinal residual that penalizes errors by label distance enforces order-consistent probabilities and improves accuracy and calibration—without extra distance-weight tuning.

In recent years, the rapid development of deep learning has led to the widespread adoption of smart healthcare. Tasks that previously required manual judgment or annotation by physicians can now be assisted by AI, helping in image classification, image segmentation, or object detection, among other applications. When using computed tomography (CT) scans to detect sarcopenia, the primary reference is the CT slices of the lumbar spine (L1–L5). Notably, when weight loss and muscle mass reduction occur, these changes are most evident in the third lumbar vertebra (L3), making it the key focus in such assessments. A systematic review of 388 published studies on CT muscle measurement found that the most commonly used technique also relied on measurements at the third lumbar vertebra [1]. Recent studies employing deep learning techniques similarly tend to assess muscle mass at the L3 vertebra [2,3,4,5,6,7]. However, in current clinical practice, when physicians examine patients, they typically generate a series of CT images that may include the twelfth thoracic vertebra (T12) and the entire lumbar spine. Physicians must manually identify the third lumbar vertebra, a time-consuming and labor-intensive process [8,9,10,11].

Classifying the third lumbar vertebra in the context of sarcopenia is an ordinal regression problem, as the lumbar vertebrae are sequentially ordered from the first to the fifth. Therefore, if deep learning methods for image classification could incorporate a loss function that considers ordinal relationships, the automated identification of the third lumbar vertebra could be more accurate. Such a model would likely outperform one that does not account for the ordinal nature of the vertebrae, ultimately saving time and effort for physicians.

## 2. Related Work

### 2.1. Ordinal Regression Based on Binary Classification

Ordinal regression is a classic problem in statistics, often encountered in tasks such as disease severity classification or age estimation, where the data has an inherent order. In the field of machine learning, ordinal regression problems are typically transformed into a series of simpler binary classification sub-problems. In 2006, Li and Lin [12] proposed a reduction framework in which, for each ordered category, assume the data categories are 1, 2, 3, …, k. A binary classifier is trained to determine whether the sample’s category is greater than k, resulting in a total of k-1 classifiers. The first classifier determines whether the data category is greater than 1, the second classifier determines whether it is greater than 2, and so on. If the first classifier output is greater than 1, the task output is 1; if the second classifier output is greater than 2, the task output is also 1; otherwise, the output is 0. The final prediction is the sum of the outputs from the k-1 classifiers, which represents the predicted category of the sample. In this paper, the Support Vector Machine (SVM) [13] classification algorithm is used for training these classifiers.

In 2016, Niu et al. [14] built upon the method proposed by Li and Lin, integrating Convolutional Neural Networks (CNNs) to solve the binary classification sub-problems, applying this approach to age estimation from facial images. In this method, the CNN has multiple output layers, each corresponding to a binary classifier. Consequently, all binary classifiers can be jointly trained within the same CNN. Since the binary classification tasks share the same hidden layer features within the CNN, the relationships between different tasks can be leveraged, thereby improving the overall model performance.

Although the approach of dividing the problem into multiple binary classifiers can address the classification of ordinal categories, each binary classification task is treated independently. This can lead to inconsistencies in the predicted probabilities across the different ordinal levels. For example, in Table 1, assume there are four ordinal categories: 1, 2, 3, and 4, and the true category for a given data point is 2. In a correct prediction scenario, as shown in Case 1, the probability of predicting category 2 should be 0.7. Due to the ordinal nature of the categories, the predicted probabilities for categories closer to 2 should be higher than those further away; hence, the probability for category 4 should be the smallest. Conversely, in an incorrect prediction scenario, as shown in Case 2, the probability for category 4 is higher than that for category 3, resulting in an inconsistency in the predicted probabilities across the ordinal levels.

### 2.2. Addressing Inconsistencies in Predicted Probabilities Across Ordinal Levels

To address the issue of inconsistent predicted probabilities across ordinal levels, Cao et al. [15] proposed the Consistent Rank Logits (CORAL) framework. This method aims to avoid increasing the complexity of training by applying weight sharing in the penultimate layer of the neural network. This ensures that all binary classification tasks in the final layer share the same weight parameters, with the only difference being the bias term for each binary classifier. For each binary classification task in the final layer, the predicted probability from the neural network is computed as shown in Equation (1). Here, $\hat{P}({y_{i}}^{\left(k\right)}=1)$ represents the output of 1 if the predicted probability of the binary classifier is greater than 0.5, and 0 otherwise. The term $g(x_{i}\;,\;W)$ denotes the sum of the outputs from all neurons in the penultimate layer, where W represents the shared weight. Since weight sharing is applied, $g(x_{i}\;,\;W)$ remains the same for each neuron in the final layer, with the only difference being the bias term $b_{k}$, which varies between neurons in the final layer. The activation function σ is a sigmoid function.

This approach demonstrates that during training, minimizing the loss function, which involves finding the optimal $W^{*}$ and $b^{*}$, will satisfy the condition $b_{1}^{*}\geq b_{2}^{*}\geq \cdots \geq b_{K-1}^{*}$. When this condition is met, the predicted probabilities will decrease as the ordinal level increases, forming a consistent relationship $\hat{P}({y_{i}}^{\left(1\right)}=1)\geq \hat{P}({y_{i}}^{\left(2\right)}=1)\geq \cdots \geq \hat{P}({y_{i}}^{\left(k-1\right)}=1)$, thereby achieving consistency in the predicted probabilities across ordinal levels. This method was applied to age estimation from facial images and demonstrated better performance compared to the previous method proposed by Niu et al. [14].

(1) $$\hat{P}({y_{i}}^{\left(k\right)}=1)=\sigma (g(x_{i}\;,\;W)+b_{k})$$

Although the CORAL method effectively addresses the issue of inconsistent predicted probabilities across ordinal levels, its use of weight sharing can impose significant constraints on the neural network, potentially reducing its performance. In 2021, Shi et al. [11] improved upon the CORAL method by removing the weight sharing requirement and proposed a new framework called Conditional Ordinal Regression for Neural Networks (CORN). This framework achieves consistency in predicted ordinal levels by utilizing the chain rule of conditional probability. The calculation of the output probability for each binary classification task in the final layer of the neural network is given by Equation (2). Here, $r_{k}$ represents the ordinal level, which has an inherent order, including $r_{1}\;,\;r_{2}\;,\;r_{3}\;,\ldots ,r_{k}$. The equation expresses the probability of the class label $y^{\left[i\right]}$ being greater than $r_{k}$, given that $y^{\left[i\right]}$ is greater than $r_{k-1}$. For example, if a dataset has ordinal categories 1, 2, 3, and 4, the first binary classifier’s $f_{k}\left(x^{\left[i\right]}\right)$ would estimate the probability that the label is greater than category 1. The second binary classifier’s $f_{k}\left(x^{\left[i\right]}\right)$ would estimate the probability that the label is greater than category 2, given that it is greater than category 1, and so on. This chain rule allows the output probabilities of each binary classifier to form a sequential relationship, as shown in Equation (3), ensuring that as the ordinal level increases, the predicted probability decreases, thereby achieving consistency in the predicted ordinal levels, as expressed in Equation (4).

(2) $$f_{k}\left(x^{\left[i\right]}\right)=\hat{P}\left(y^{\left[i\right]}>r_{k}\;|\;y^{\left[i\right]}>r_{k-1}\right)$$

(3) $$\hat{P}\left(y^{\left[i\right]}>r_{k}\right)=\prod_{j=1}^{k}\;f_{j}\left(x^{\left[i\right]}\right)$$

(4) $$\hat{P}\left(y^{\left[i\right]}>r_{1}\right)\geq \hat{P}\left(y^{\left[i\right]}>r_{2}\right)\geq ...\geq \hat{P}\left(y^{\left[i\right]}>r_{K-1}\right)$$

Moreover, CORN estimates the series of conditional probabilities using conditional training subsets, as the following Equation (5).

(5) $$\begin{array}{l} S_{1}:all\{(x^{\left[i\right]},y^{\left[i\right]})\},for\;i\in \text{\{}1,\ldots .,\;N\text{\}},\\ S_{2}:\{(x^{\left[i\right]},y^{\left[i\right]})|y^{\left[i\right]}>r_{1}\},\ldots .S_{K-1}\text{:}\{(x^{\left[i\right]},y^{\left[i\right]})|y^{\left[i\right]}>r_{k-2}\} \end{array}$$

where $S_{1}$ represents the entire training set, used by the first binary classifier to determine the probability that a sample’s category is greater than 1. $S_{2}$ represents all samples with categories greater than 1, used by the second binary classifier to determine the probability that a sample’s category is greater than 2, given that it is greater than 1, and so forth [11]. Although the CORN method outperforms the CORAL method, its primary drawback is that it requires modifications to the structure of the model’s output layer before training.

### 2.3. Other Neural Network-Based Methods for Ordinal Regression

In addition to the approach of extending ordinal regression into multiple binary classification tasks, as discussed earlier, there are other methods that do not rely on this extension. In 2019, Diaz and Marathe [16] proposed a method using soft labels to address the ordinal regression problem. Soft labels account for the ordered nature of the categories. For example, consider a dataset with categories 1, 2, 3, 4, and 5 in an ordinal sequence. If the true label for a data point is 3, its one-hot encoding would be [0, 0, 1, 0, 0]. When predicting this data point as category 1 (one-hot encoded as [1, 0, 0, 0, 0]), the loss would be the same as if it were predicted as category 2 (one-hot encoded as [0, 1, 0, 0, 0]). However, intuitively, the loss for predicting category 1 should be greater than for predicting category 2, since category 1 is farther from the true label of 3. To address this, the authors proposed transforming one-hot encodings into soft labels. For example, if the true label is 3, the one-hot encoding [0, 0, 1, 0, 0] could be transformed into a soft label such as [0, 0.3, 0.4, 0.3, 0]. This way, the loss for predicting category 1 would be greater than the loss for predicting category 2, reflecting the ordinal nature of the labels.

**Figure 1:**
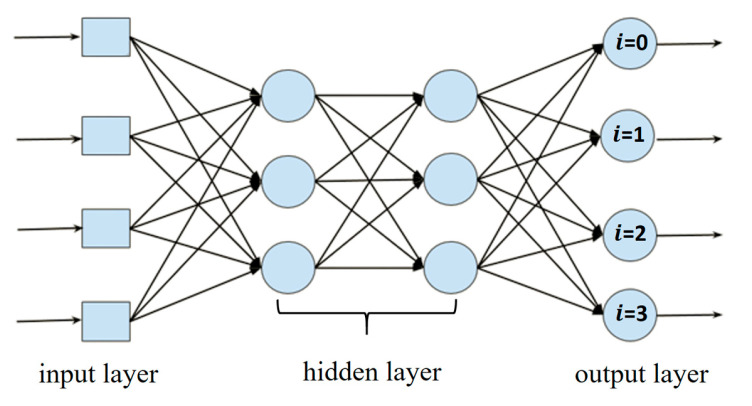
Classification network.

In 2022, Polat et al. [17] proposed a new loss function called Class Distance Weighted Cross-Entropy (CDW-CE), which is based on the traditional cross-entropy loss function. The formula for the cross-entropy loss is given in Equation (6), where *i* represents the index of the category in the output layer. As illustrated in Figure 1, if a classification network is trained with four categories as input, the output will also have four categories, each with its corresponding predicted probability. The category with the highest probability is considered the predicted result. Here, *i* = 0 represents the first category, and so on. In the formula, $y_{i}$ denotes the one-hot encoding of the *i*-th category, while ${\hat{y}}_{i}$ represents the predicted probability for the *i*-th category. The overall meaning of the formula is that cross-entropy is calculated by multiplying the one-hot encoding of each category by the logarithm of its predicted probability, and then summing these values. Since the one-hot encoding for the ground truth category is 1, and for the non-ground truth categories it is 0, the cross-entropy loss function focuses solely on the predicted probability of the true category, applying a penalty through the log function. Therefore, the formula can be simplified to $-log{\hat{y}}_{c}$, where ${\hat{y}}_{c}$ represents the predicted probability of the true category.

(6) $$\mathrm{Cross}\;\mathrm{Entropy}=-\sum_{i=0}^{N-1}\;y_{i}\times log{\hat{y}}_{i}=-log{\hat{y}}_{c}$$

(7) $$\mathrm{CDW}\text{-}\mathrm{CE}=-\sum_{i=0}^{N-1}\;log(1-{\hat{y}}_{i})\times {\left|i-c\right|}^{a}$$

The CDW-CE formula, as shown in Equation (7), modifies the standard cross-entropy loss function by incorporating the predicted probabilities of negative classes. Instead of only considering the predicted probability of the true class, the CDW-CE formula also takes into account the log of the predicted probabilities for the negative classes, modified to $1-{\hat{y}}_{i}$. Since the categories have an ordinal relationship, the formula further multiplies by $\left|i-c\right|$, which represents the distance between the index *i* of the predicted category and the index *c* of the true category. This adjustment introduces a penalty based on how far the predicted category is from the true category. The parameter *a* is a hyperparameter that controls the strength of this distance penalty, where a larger *a* results in a stronger penalty.

**Figure 2:**
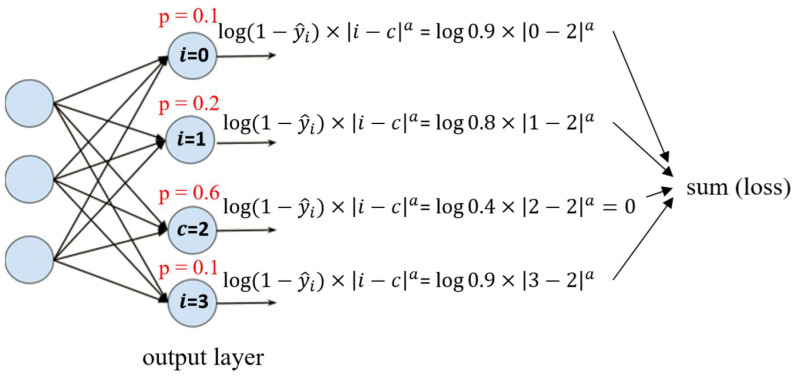
Diagram of the CDW-CE formula.

Figure 2 illustrates this concept. Here, *P* represents the predicted probability for each category, and if the true category index is 2, then for the true category, $\left|2-2\right|=0$, resulting in a loss of 0, since the loss for the true category should be minimized. The final loss is the sum of the penalized losses for the negative categories, weighted by their distance from the true category.

This loss function was applied in a study estimating the severity of ulcerative colitis. The experiment used three CNN architectures with pre-trained models: ResNet18 [18], Inception-v3 [19], and MobileNet-v3-large [20]. The results showed that this method not only outperformed the standard cross-entropy loss function but also surpassed the CORN method proposed by Shi et al. [11]. Furthermore, the experiment utilized the CAM [21] technique for neural network visualization to highlight the areas of the images that the network focused on for its predictions. These highlighted areas were then evaluated by professional physicians, and the statistical results indicated that the areas identified by the network trained with CDW-CE were more consistent with the physicians’ assessment criteria compared to those identified using the standard cross-entropy loss function.

However, despite the improvements offered by the CDW-CE method over previous studies, it also has certain limitations. First, since the parameter *a* in the CDW-CE formula (Equation (7)) is an exponent and is not fixed, we cannot directly determine the optimal value of *a* during the training process. The appropriate strength of the penalty must be manually tested to achieve the best training results. This issue was also mentioned in the paper, where they tested values of *a* ranging from 1 to 10, conducting cross-validation for different models to select the optimal *a* value. When training the ulcerative colitis dataset with three different models, the optimal *a* value is found to be around 6. Once the *a* value exceeds the optimal point, the larger the value of *a*, the more unstable the training becomes, leading to an increase in standard deviation and a significant decrease in QWK (Quadratic Weighted Kappa) [17]. Therefore, a higher penalty strength is not necessarily better. Additionally, as *a* increases, the training time also becomes longer. Their study also emphasizes the need to try different *a* values, as the optimal value may vary depending on the dataset, the number of classes, or the model architecture used. Overall, their study identifies two main limitations:

1. The exponent *a* in the CDW-CE formula requires manual tuning of different values, making the process more time-consuming. Additionally, as *a* increases, the penalty intensity also increases, leading to higher loss values and longer training times.
2. Through cross-validation training, larger *a* values result in less stable training, with a significant increase in standard deviation.

## 3. Materials and Methods

### 3.1. Research Framework

**Figure 3:**
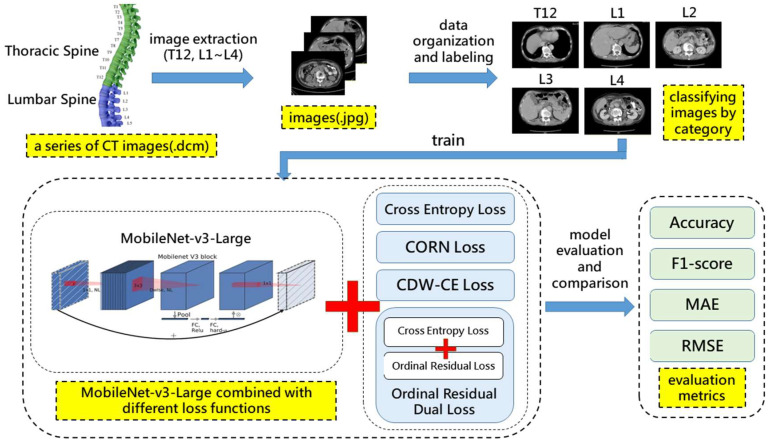
Research Framework Diagram.

Figure 3 illustrates the research framework of this retrospective study. Initially, when a patient undergoes a CT scan, a series of CT images in .dcm format is produced. The .dcm files are multi-slice, continuous CT images that form a 3D representation. The physician then extracts the required dataset from this series of images, including the twelfth thoracic vertebra (T12) and the first to fourth lumbar vertebrae (L1–L4). Vertebral levels (T12–L4) served as the reference standard for level classification; expert labels by credentialed radiologists are widely used for vertebral identification in imaging AI and constitute the de facto reference standard. Following this, the data is organized and classified, with CT images from multiple patients categorized accordingly. An experienced physician independently annotated the vertebral levels. Test labels were annotated by the same physician; the annotator was blinded to model outputs. Inter- and intra-rater reliability were not assessed because a single experienced physician produced all reference labels. Once the dataset is prepared, it is fed into the MobileNet-v3-Large network, and experiments are conducted using different loss functions. The backbone is MobileNet-v3-Large initialized with ImageNet-1K pretrained weights (torchvision); no other pretraining was used. Our method employs the Ordinal Residual Dual Loss to compute the losses, which combines the cross-entropy loss function with the Ordinal Residual Loss. Adam was the optimizer, and early stopping halted training when performance gains ceased. Finally, the results are evaluated and compared using relevant metrics.

### 3.2. Ordinal Residual Dual Loss

The approach of the Ordinal Residual Dual Loss combines the cross-entropy loss function and the Ordinal Residual Loss. It is referred to as “dual” because of the combination of these two loss functions. The cross-entropy loss function is given by Equation (6), and the Ordinal Residual Loss is described in Equation (10). Here, *i* represents the class index, and $d_{i}$ denotes the difference between the predicted probability and the target probability for a specific class, as defined in Equation (9). $P_{i}$ is the predicted probability for a particular class, while $O_{i}$ is the target probability for that class. The method for calculating the target probability is described in Equation (8), where *c* is the index of the true class, and *N* is the total number of classes.

**Figure 4:**
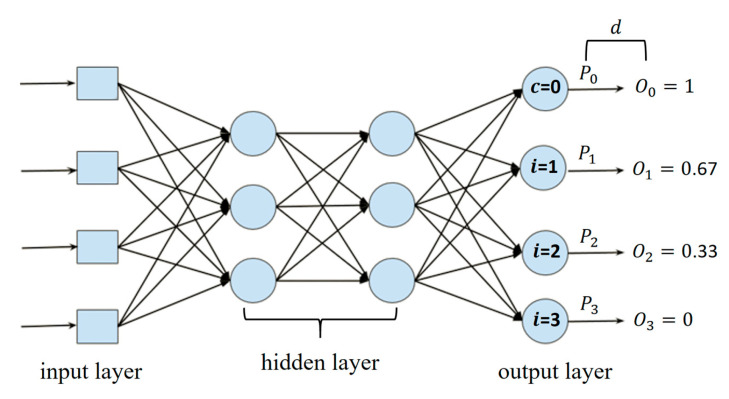
Diagram of the Ordinal Residual Loss formula.

The Ordinal Residual Loss is designed to consider the distance between the predicted probability and the target probability when calculating the loss. The residual refers to the difference between these two probabilities, as illustrated in Figure 4. Suppose there are four classes, and the network predicts a true class index *c* (with *c* = 0). The predicted probabilities for the first class are $P_{0}$, for the second class $P_{1}$, and so on. We aim for the predicted probabilities to reflect the ordinal nature of the regression. Therefore, we construct a set of ordinal target probabilities $O_{i}$. The goal is for the predicted probabilities to gradually approach the target probabilities, thus achieving ordinal regression. To this end, we calculate the difference $d_{i}$ between the predicted and target probabilities. The Ordinal Residual Loss is then used to compute the loss, where a smaller difference $d_{i}$ results in a lower loss, and a larger difference results in a higher loss. By combining the Ordinal Residual Loss with the cross-entropy loss function, the Ordinal Residual Dual Loss provides feedback for the network’s training process.

(8) $$O_{i}=1-\left(\frac{\left|i-c\right|}{N-1}\right)$$

(9) $$d_{i}=\left|P_{i}-O_{i}\right|$$

(10) $$Ordinal\;Residual\;Loss=-\sum_{i=0}^{N-1}\;log(1-d_{i})$$

### 3.3. CT Image Dataset

#### 3.3.1. Dataset Description

**Figure 5:**
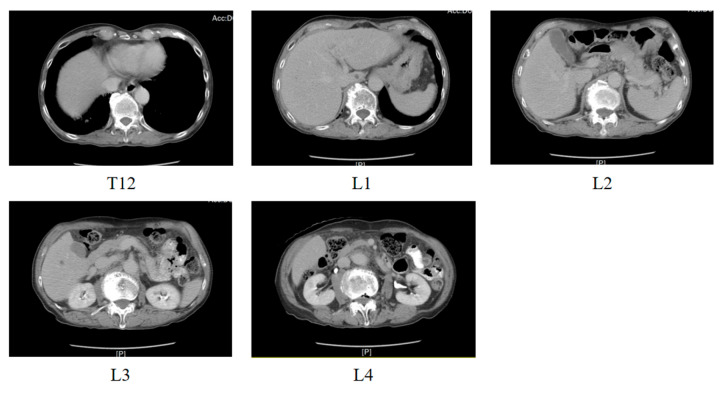
T12, L1~L4 from a patient.

This study utilizes a dataset of CT image slices provided by Dalin Tzu Chi Hospital, Chiayi (IRB number: B11102012). The dataset comprises images from 84 patients, each including 4 to 6 CT scans of the twelfth thoracic vertebra (T12) and the first through fourth lumbar vertebrae (L1–L4), totaling 2512 CT images. The dataset included adults undergoing abdominopelvic CT with contiguous T12–L4 coverage and adequate image quality, excluding those with prior lumbar surgery, vertebral anomalies or fractures, severe motion artifacts, or incomplete coverage. As shown in Table 2, the dataset includes 474 images of the twelfth thoracic vertebra, 482 images of the first lumbar vertebra, 510 images of the second lumbar vertebra, 548 images of the third lumbar vertebra, and 498 images of the fourth lumbar vertebra. Figure 5 below illustrates the CT scans of the twelfth thoracic vertebra and the first through fourth lumbar vertebrae from one patient.

#### 3.3.2. Dataset Partitioning and Data Preprocessing

In the CT image dataset, a series of CT scans from a single patient (T12, L1~L4) is continuous, but there are differences between the CT scans of different patients. Therefore, in practice, we train the model on CT scans from multiple patients to predict the classification of CT scans from unseen patients. For this study, the dataset was split based on patient count into an 8:2 ratio, with 67 patients’ data used for the training set and 17 patients’ data for the test set. Since the number of CT scans per series for each patient is approximately the same, the overall dataset split also roughly adheres to the 8:2 ratio, as detailed in Table 3. The independent test set comprised all consecutive eligible cases during the study period; no a priori power calculation was performed due to the retrospective design. Only imaging data were available; no demographic/clinical variables were provided, so per-partition characteristics are not reported. Additionally, ten-fold cross-validation was employed for training in this study.

DICOM headers were scrubbed and all direct identifiers removed prior to analysis; only anonymized study IDs were used. For each patient, a clinically acquired series spanning T12 through L4 was available, and the slices within each individual exam were contiguous. Each CT slice was loaded and standardized in format, then resized to the model’s input resolution while preserving the original aspect ratio; when aspect ratios differed, padding was applied to maintain anatomical proportions. Pixel intensities were normalized according to the backbone’s pretraining conventions to reduce variability from acquisition settings. Finally, centered and boundary cropping removed black margins and scanner annotations, focusing the field of view on the vertebral body and adjacent paraspinal musculature.

## 4. Results

The software used in this study was developed with Python 3.7, utilizing the PyTorch 1.12.0 framework for deep learning training. In our experiments, the dataset was split into an 8:2 ratio based on patient count for ten-fold cross-validation. We employed the pre-trained MobileNet-v3-Large model for training. For all methods except the CDW-CE method, the learning rate was set to e−4. In the case of the CDW-CE method, due to the presence of the *α* parameter, larger values of *α* increase the loss, making the model harder to converge and more unstable. As a result, the loss can sometimes become NaN during training. To address this, we reduced the learning rate for the CDW-CE method to e−5. The Adam optimizer was used, along with Early Stopping to halt training when model performance no longer improved. Training used the Adam optimizer with early stopping (patience 10, max 1000 epochs), batch size 8, weight decay 1 × 10^−4^, and standard label-preserving augmentations (resize to 224 × 224 with 3-channel ImageNet normalization). Early stopping monitored validation performance on the held-out fold in each 10-fold cross-validation round, halting training once improvement ceased and retaining the best-epoch checkpoint, with all splits performed at the patient level using an 8:2 train–test ratio (no patient overlap). We compared the proposed Ordinal Residual Dual Loss function method against the standard cross-entropy loss function and other methods from previous literature.

The experimental results are shown in Table 4. When comparing our method to using the cross-entropy loss function alone, our method demonstrated better performance, with accuracy improving from 0.8467 to 0.8726, MAE decreasing from 0.1652 to 0.1373, and RMSE decreasing from 0.4435 to 0.4015. Additionally, we applied a two-sample *t*-test to assess whether the accuracy difference between the two methods was statistically significant. The calculated *t*-value was −3.1978, with a degree of freedom of 17.17, and a *p*-value of 0.0052. Given that the *p*-value is less than 0.05 at a significance level of *α* = 0.05, we can reject the null hypothesis, indicating that our method is significantly better than using the cross-entropy loss function alone. We pre-specified CE vs. the proposed loss as the primary comparison and used a two-sample *t*-test over fold accuracies (*t* = −3.1978, df = 17.17, *p* = 0.0052; *α* = 0.05); other baselines are reported descriptively as mean ± SD without inferential testing.

**Figure 6:**
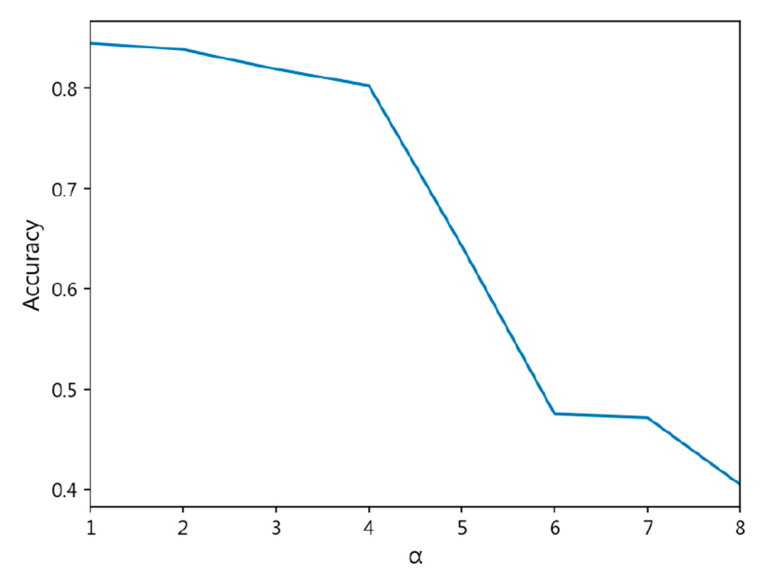
Accuracy of MobileNet-v3-Large with Different *α* Values Using CDW-CE Loss.

**Figure 7:**
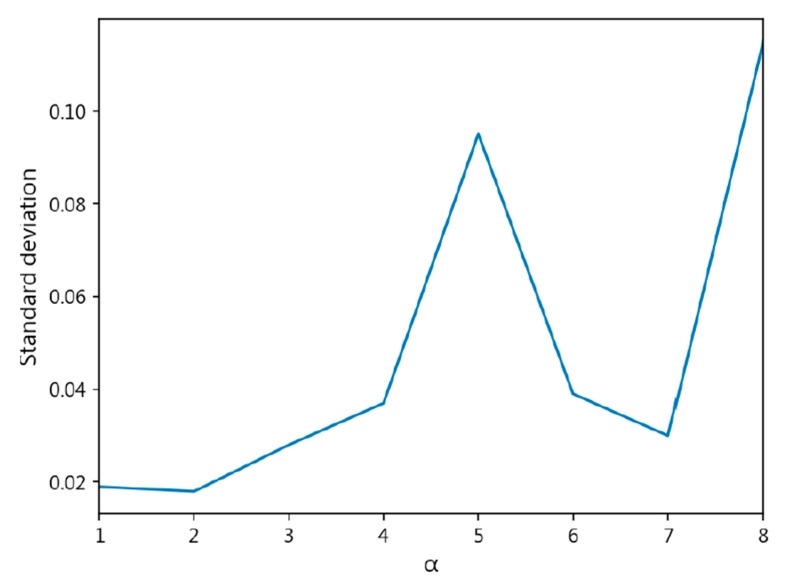
Standard Deviation of Accuracy with Different *α* Values Using CDW-CE Loss on MobileNet-v3-Large.

However, the model using the CORN Loss and CDW-CE methods did not outperform the cross-entropy loss function, indicating that our method is superior to previous approaches. Figure 6 illustrates the accuracy changes of MobileNet-v3-Large at different *α* values, showing a rapid decline in accuracy after α=4, with loss values becoming NaN at α=9 and α=10, making the model more difficult to converge. Figure 7 presents the changes in the standard deviation of accuracy at different *α* values, where the standard deviation increases sharply as *α* becomes larger. Overall, our method, when tested on MobileNet-v3-Large, still outperforms other methods.

As shown in Table 5, across three ImageNet-pretrained backbones trained under identical settings with Ordinal Residual Dual Loss, MobileNet-v3-Large achieves the highest Accuracy/F1 (0.8726 ± 0.020/0.8726 ± 0.019) and the lowest MAE (0.1373 ± 0.022), ResNet-34 is comparable on Accuracy/F1 (0.8702 ± 0.026/0.8706 ± 0.026) and yields the best RMSE (0.3918 ± 0.050), while EfficientNetV2-S remains close on Accuracy/F1 (0.8718 ± 0.022/0.8699 ± 0.021) with slightly larger errors (MAE 0.1429 ± 0.024; RMSE 0.4189 ± 0.041). These differences fall within roughly one standard deviation, indicating the conclusions are not backbone-dependent and that ORDL provides stable, consistent performance. Accordingly, we retain MobileNet-v3-Large as the primary model for its slight edge in accuracy/MAE.

## 5. Discussion

In this study, we utilized the MobileNet-v3-Large network model to classify a series of CT scans for patients, including the twelfth thoracic vertebra and the first through fourth lumbar vertebrae. The data categories exhibit an ordinal relationship, so the primary focus of this study is to account for the hierarchical order among the classes during the image classification process. To achieve this, we proposed the Ordinal Residual Dual Loss function, which combines the cross-entropy loss function with the Ordinal Residual Loss. By calculating the distance between the predicted and target probabilities and then computing the loss, we aim for the predicted probabilities to gradually approach a set of ordinal target probabilities, thereby achieving ordinal regression. The final experimental results demonstrate that our method outperforms both the cross-entropy loss function alone and methods from previous literature.

Overall, this study achieves two main contributions: First, we propose the Ordinal Residual Dual Loss function, which, when compared to the cross-entropy loss function, improves the training accuracy of MobileNet-v3-Large from 0.8467 to 0.8726, reduces the MAE from 0.1652 to 0.1373, and reduces the RMSE from 0.4435 to 0.4015. The results also surpass those of previous studies. Second, while previous methods such as CORN require manual adjustment of the model’s output structure before training, and CDW-CE requires manual tuning of different *α* parameters—with larger *α* values leading to longer and more unstable training—our method does not require any manual parameter adjustments.

In the real world, many prediction problems involve categories with ordinal relationships. Sarcopenia, a common disease among the elderly, can be diagnosed using lumbar spine scans, where the vertebrae are sequentially ordered. This makes it an ordinal regression problem. By applying deep learning-based image classification methods, we can assist physicians in quickly identifying the third lumbar vertebra, thereby saving time and making the diagnostic process more efficient for patients.

There are two potential areas for improvement in this study. First, during CT scans, the output may include an entire section of the thoracic and lumbar spine. In this study, the dataset was curated by physicians to include only the twelfth thoracic vertebra and the first through fourth lumbar vertebrae for training. In the future, the dataset could be expanded to include additional categories, allowing for training on the entire spine, which would enable the model to learn from a broader range of image data and enhance its recognition capabilities.

The second improvement involves the training method. In this study, we trained the model by mixing CT images from different patients across various categories. However, each patient’s lumbar CT images are unique, with a series of images from a single patient being continuous and similar. A future improvement could involve training the model using a series of CT images from the same patient at once, rather than individual images. This approach would allow the model to learn from a set of images as a coherent series from a single patient, making the training process more aligned with practical applications.

## Figures and Tables

**Table 1:** The case of inconsistencies in the predicted probabilities.

Class	Predicted Probabilities	
	Case 1 (Correct)	Case 2 (Incorrect)
1	0.2	0.1
2	0.7	0.6
3	0.1	0.1
4	0	0.2

**Table 2:** The number of CT image datasets.

	All	T12	L1	L2	L3	L4
Number of Datasets	2512	474	482	510	548	498

**Table 3:** Dataset Partitioning.

	Number of Patients	Number of Datasets	T12	L1	L2	L3	L4
**Training dataset**	67	2003	383	380	408	441	391
**Testing dataset**	17	509	91	102	102	107	107

**Table 4:** Experimental Results of MobileNet-v3-Large.

MobileNet-v3-Large		Accuracy	F1-Score	MAE	RMSE
**Cross Entropy Loss**		0.8467 ± 0.016	0.8464 ± 0.017	0.1652 ± 0.025	0.4435 ± 0.072
**CORN**		0.7958 ± 0.051	0.7950 ± 0.050	0.2125 ± 0.057	0.4747 ± 0.074
**CDW-CE**	α = 1	0.8447 ± 0.019	0.8441 ± 0.021	0.1652 ± 0.024	0.4309 ± 0.043
	α = 2	0.8384 ± 0.018	0.8402 ± 0.017	0.1716 ± 0.016	0.4390 ± 0.018
	α = 3	0.8189 ± 0.028	0.8192 ± 0.027	0.1939 ± 0.026	0.4700 ± 0.029
	α = 4	0.8025 ± 0.037	0.8013 ± 0.037	0.2054 ± 0.036	0.4690 ± 0.037
	α = 5	0.6429 ± 0.095	0.5906 ± 0.142	0.3674 ± 0.096	0.6192 ± 0.080
	α = 6	0.4753 ± 0.039	0.3532 ± 0.029	0.5338 ± 0.038	0.7426 ± 0.026
	α = 7	0.4717 ± 0.030	0.3481 ± 0.022	0.5386 ± 0.032	0.7479 ± 0.026
	α = 8	0.4049 ± 0.115	0.2839 ± 0.112	0.8575 ± 0.614	1.1068 ± 0.700
	α = 9	NaN			
	α = 10	NaN			
**Ordinal Residual Dual Loss**		**0.8726 ± 0.020**	**0.8726 ± 0.019**	**0.1373 ± 0.022**	**0.4015 ± 0.040**

**Table 5:** Experimental Comparison of Different Backbones.

Ordinal Residual Dual Loss	Accuracy	F1-Score	MAE	RMSE
**MobileNet-v3-Large**	0.8726 ± 0.020	0.8726 ± 0.019	0.1373 ± 0.022	0.4015 ± 0.040
**ResNet-34**	0.8702 ± 0.026	0.8706 ± 0.026	0.1377 ± 0.030	0.3918 ± 0.050
**EfficientNetV2-s**	0.8718 ± 0.022	0.8699 ± 0.021	0.1429 ± 0.024	0.4189 ± 0.041

## Data Availability

The original contributions presented in this study are included in the article. Further inquiries can be directed to the corresponding author.
